# Assessment of Food Masticatory Capability with Clear Aligners

**DOI:** 10.3390/dj12070217

**Published:** 2024-07-15

**Authors:** Luca Levrini, Nicola Giannotta, Rodolfo Francesco Mastrapasqua, Davide Farronato, Vittorio Maurino, Alessandro Deppieri, Federico Tasquier, Stefano Saran

**Affiliations:** 1 Department of Human Sciences, Innovation and Territory, School of Dentistry, Postgraduate of Orthodontics, University of Insubria, 21100 Varese, Italy; luca.levrini@uninsubria.it (L.L.); ngiannotta@studenti.uninsubria.it (N.G.); adeppieri@studenti.uninsubria.it (A.D.); tasquierfederico@gmail.com (F.T.); 2 ENT Department, Rivoli Hospital, ASL TO3, 10098 Rivoli, Italy; rfmastrapasqua@aslto3.piemonte.it; 3 Department of Medicine and Surgery, School of Dentistry, University of Insubria, 21100 Varese, Italy; davide.farronato@uninsubria.it (D.F.); vittorio.maurino@uninsubria.it (V.M.)

**Keywords:** aligner, chewing, food, orthodontic appliances, orthodontic, eating, compliance, fitting, retention

## Abstract

Nowadays, aligners represent a possible therapeutical approach that combines both esthetic and function in order to address dental malocclusion. However, they require a significant level of compliance from the patient. According to the manufacturer, at least 22 h of wearing a day is demanded to reach the optimal therapeutical level; hence, aligners can only be removed during meals. Patients’ compliance might increase and the duration of the treatment might decrease if they were allowed to eat with aligners on. The idea of patients keeping the aligners on during meals has been contemplated, not only to favor patients’ compliance but also treatment effectiveness. This study aims to assess the degree of chewing difficulty that aligners cause when eating certain kinds of food and the quantity of residue left. Material and Methods: A questionnaire titled “Questionnaire for the Assessment of Masticatory Function with Aligners” was administered using Google Forms to 240 patients in treatment with clear aligners. The survey was validated through the reliability test using the test–retest method. This method had a higher correlation coefficient of 0.9 across all items (with a cutoff of 0.8) with statistical significance, and an excellent internal correlation coefficient (α > 0.9). The statistical analysis performed consisted of descriptive analysis, frequencies, percentages, Pearson’s correlation test and Friedman’s test. Results: Pearson’s test showed a statistically significant correlation between all items except between meat or clams and yogurt or ice cream and with mozzarella or soft cheese regarding food chewing difficulties. Pearson’s test showed a statistically significant correlation between all items regarding food residues. A total of 69.2% of the cohort reported some movements of aligners during mastication. In total, 88.3% of them affirmed not to have perceived deformations or breakage of aligners during chewing. Furthermore, 79.2% of them declared that they would continue to eat if eating with aligners was proven to speed up treatment. Conclusions: Wearing clear aligners while chewing foods such as yogurt, ice cream, soft cheese, bread, rice, etc., can be possible and can help shorten the duration of orthodontic treatment, benefiting both the patient and the orthodontist. However, further research using qualitative methods is needed to understand the barriers and facilitators to chewing food with aligners.

## 1. Introduction

Clear aligner therapy (CAT), similar to fixed appliance systems, uses a variety of appliances with different construction techniques, modes of action, and appropriateness for treating certain malocclusions. While all of these systems employ clear aligners made of thermoformed plastic that cover most or all of the teeth, there are notable variations that have an impact on how well these systems work to treat a variety of orthodontic issues [1]. Aligners are nowadays a possible therapeutical approach that can combine esthetic and function in addressing dental malocclusion. According to what is demonstrated in the literature, the main reason patients seek orthodontic treatments is to improve their esthetics, and consequently, they are looking for a therapy that impacts their appearance as little as possible while in progress. Clear aligners, in contrast to other orthodontic methods, can be removed, facilitating regular cleaning of the devices, maintaining home hygiene, and eating comfortably. CAT has several advantages besides esthetics and function: it allows for a better domiciliary hygiene and consequently helps to prevent periodontal related diseases, and it has a lower incidence of cavities and a superior preservation of surface enamel [2,3]. Furthermore, the level of comfort with aligners is proven to be higher than with fixed devices [4]. The downside is the need of compliance; CAT requires, according to manufacturers, approximately 20–22 h per day and to reach optimal effectiveness, it requires continuous and consistent wear over a 24 h period [5,6].

However, clear aligners are often taken out of the mouth during meals and oral hygiene practices, leading to frequent interruptions in their effectiveness.

The actual guidelines indicate that aligners need to be removed only for eating and for drinking hot beverages that may cause warping or staining or beverages that contain sugar, and when brushing and flossing [7].

Considering the necessity to reach the ideal 24 h, the possibility to masticate with the clear aligner on should be analyzed through chemical and physical tests on aligners and with a survey that highlights the difficulties to chew. Removing the aligners leads to constant deformation; therefore, it affects the efficacy of the treatment. It is reasonable to suppose that eating and drinking with them on can reduce the length of therapy [8].

A possible factor that can influence chewing is the consistency of food, which can alter the capability to keep aligners steady during meals.

Today’s aligners have an average thickness of 0.6 mm, resulting in a change in the patient’s vertical dimension of about 1.2 mm [9].

According to the literature, the insertion of an orthodontic device that increases the occlusal vertical dimension does not have a significant influence on the masticatory performance of the subjects, which indicates that the chewing function immediately adapts to the changed occlusal condition [10,11].

Instead, a flatter occlusal table requires more force to allow the cusps to grind the food, which could lead to a decrease in masticatory efficiency [11].

Masticatory function affects a variety of orofacial components, including teeth, salivary glands, nerves, and masticatory muscles [12]. The patient’s subjective appraisal of their ability to chew or the results of objective testing methods can be used to evaluate their chewing function (chewing efficiency) [13,14].

In the literature, validated surveys analyzing the chewing efficiency with aligners are yet to be formulated and no study to this day has evaluated the change in aligners after consuming food. However, a few studies affirm that the physical consistency of aligners is not altered by foods [15]. 

The aim of our study is to assess the level of difficulty to chew certain foods and the amount of residue left using orthodontic aligners.

## 2. Material and Methods

### 2.1. Study Participants

A questionnaire titled “Questionnaire for the Assessment of Masticatory Function with Aligners” was administered electronically using Google Forms to 240 patients in treatment with clear aligners from March 2023 to October 2023. Through a subjective analysis, certain parameters were determined for the sample in question. Back-translation was performed with the assistance of a native English speaker. The questionnaire was translated from Italian to English and then back to Italian by the translator to assess any potential issues. The questionnaire underwent validation before spreading. The research was approved and conducted according to the ethics committee n. 0111335 of “Università degli Studi dell’Insubria”, Varese, Como, Italy. Patients were requested to complete the questionnaire voluntarily, with no payment or other incentives provided. The questionnaire was requested to be filled out voluntarily by patients; there was no payment or other incentive. The survey was created especially for this investigation. 

The inclusion criteria were as follows: patients undergoing an aligner treatment with an average level of compliance, absence of TMDs, without referred chewing problems.

The exclusion criteria were as follows: absence of compliance.

All respondents gave their informed consent and accepted the privacy policy for the protection of personal information before they could participate. There was no collection of personally identifying information, and the data were simply examined in aggregate form. All the replies were gathered anonymously using Google Form. There were no identifiers in the resultant data file that were utilized for analysis, including IP addresses, email addresses, or other electronic identifiers. This study adhered to the principles outlined in the Declaration of Helsinki, ensuring ethical considerations were followed.

### 2.2. Study Design

This study is a cross-sectional one.

A list of foods was presented, and patients were asked if they experienced any difficulties while chewing with the aligners and whether they noticed any residue in their mouth. These two parameters were assessed on a scale ranging from 0 to 10. In this survey 0 was an indicator of absence of difficulty to chew the food indicated and the lack of residues after eating and 10 as maximum of difficulty and presence of residues. The adherence of aligners was assessed on a scale ranging from 0 to 5, where 0 is the indicator of minimum and 5 the maximum.

This survey was validated with test–retest. The test–retest method had a higher correlation coefficient of 0.9 across all items (the cutoff was 0.8) with statistical significance, and there was an excellent internal correlation coefficient with an alpha greater than 0.9 (Table 1 and Table 2).

The questionnaire includes the following foods: bread, yogurt or ice cream, rice, meat or clams, boiled vegetables, pasta, pizza, chewing gum, biscuits or breadsticks or crackers, fish, meatballs or hamburgers, processed meats or ham, mozzarella or soft cheese, chips or popcorn or nuts or peanuts, salad, and sandwiches.

The post hoc power of the study was calculated based on the main outcome of perceiving movements of aligners during chewing. 

In the sample considered, 30.8% affirmed to perceive movements. In the literature, there are no studies that analyze this aspect in a very specific way between patients; so, we considered a general percentage of 50%. To achieve 80% power with an alpha set at 0.05, the sample size was calculated at 51 (Formula (1)).

**Formula (1). Power of the study**

(1) $$N={\frac{0.5*0.5\left\{1.96+0.84\sqrt[{2}]{\frac{0.308*0.692}{0.5*0.5}}\right\}}{{\left(0.308-0.5\right)}^{2}}}^{2}$$

To the question “Did you notice some movements of the aligners during chewing?”, 30.8% affirmed to perceive some movements of aligners, while 69.2% denied that (Table 1).

To the question “Have the aligners ever broken or deformed during chewing?”, 10.8% affirmed to perceive deformations or breakages of aligners, while 88.3% denied that (Table 1).

To the question “If you feel food residues after chewing, is drinking enough to remove them?”, 47.5% affirmed that it is enough, while 52.5% denied that (Table 1).

To the question “Did you notice any difference in the length of your lunch with and without aligners?”, 50% affirmed that it is notable, while 49.6% denied that (Table 1).

To the question “If eating with aligners was proven to speed up treatment, would you continue to eat with aligners?”, 79.2% affirmed that they would, while 20.8% would not (Table 1).

The variables analyzed in the questionnaire are listed in the table (Table 3).

### 2.3. Statistical Analysis

The statistical analysis performed consisted of descriptive analysis, frequencies, percentages, Pearson’s correlation test and Friedman’s test. 

The parameters were tested for normality with Shapiro–Wilk test. Friedman’s test was used for repeated measures analysis of variance by ranks.

Pearson’s test (two tailed) was used to test possible correlations between items.

The *p* and alpha value are set at 0.05, adapted with Bonferroni correction.

## 3. Results

The sample consisted of 132 females (56.7%) and 101 males (43.3%), and 7 respondents did not specify their sex.

The patients were in an age range between 12 and 61 years old with a greater frequency in the range 21–31 years old and the average age was 30 (Table 4). Regarding the educational background, 92 had a diploma, 112 had a degree, 1 had an elementary license and 29 had a middle school license.

The patients of this study had a various number of attachments in a range between 0 and 28. The average of attachments that patients had is 10.07 (Table 4). It is remarkable that 31.4% had no attachments (65 patients). 

The patients reported an average of 4.59 regarding the tightness of the aligners (Table 4).

Considering the difficulty to chew and the residues from 0 to 10 (Table 5 and Table 6):

1. “Bread” was 3.75 for difficulty and 3.62 for residues.
2. “Yogurt or ice cream” was 2.13 for difficulty and 1.86 for residues.
3. “Rice” was 3.08 for difficulty and 3.10 for residues.
4. “Meat or clams” was 5.10 for difficulty and 4.25 for residues.
5. “Boiled vegetables” was 2.88 for difficulty and 3.20 for residues.
6. “Pasta” was 3.48 for difficulty and 3.27 for residues.
7. “Pizza” was 4.18 for difficulty and 3.83 for residues.
8. “Chewing gum” was 5.84 for difficulty and 5.31 for residues.
9. “Biscuits or breadsticks or crackers” was 3.30 for difficulty and 4.65 for residues.
10. “Fish” was 3.04 for difficulty and 3.02 for residues.
11. “Meatballs or hamburgers” was 3.30 for difficulty and 3.37 for residues.
12. “Cured meat” was 4.09 for difficulty and 3.38 for residues.
13. “Mozzarella or soft cheese” was 2.46 for difficulty and 2.72 for residues.
14. “Chips or popcorn or nuts or peanuts” was 4.11 for difficulty and 5.37 for residues.
15. “Salad” was 4.01 for difficulty and 3.93 for residues.
16. “Sandwiches” was 3.39 for difficulty and 3.49 for residues.

To the question “Did you notice some movements of the aligners during chewing?”, 30.8% affirmed to perceive some movements of aligners, while 69.2% denied that (Table 1).

To the question “Have the aligners ever broken or deformed during chewing?”, 10.8% affirmed to perceive deformations or breakages of aligners, while 88.3% denied that (Table 1).

To the question “If you feel food residues after chewing, is drinking enough to remove them?”, 47.5% affirmed that it is enough, while 52.5% denied that (Table 1).

To the question “Did you notice any difference in the length of your lunch with and without aligners?”, 50% affirmed that it is notable, while 49.6% denied that (Table 1).

To the question “If eating with aligners was proven to speed up treatment, would you continue to eat with aligners?”, 79.2% affirmed that they would, while 20.8% would not (Table 1).

Pearson’s test was used to determine the correlation between food chewing difficulties; the results showed a significant correlation between all items (*p* < 0.01) except between meat or clams and yogurt or ice cream and with mozzarella or soft cheese (*p* > 0.01) (Table 7).

Pearson’s test was used to determine the correlation between food residues; the results showed a significant correlation between all items (*p* < 0.01) (Table 8). 

Friedman’s test was used to evaluate the possible differences between the correlated items; the results showed significant statistical differences between the levels of difficulties and the amount of residue of different items (*p* < 0.05).

## 4. Discussion

Nowadays, the advice given by most orthodontists and the leading manufacturer of aligner is to avoid eating when wearing aligners. 

This is because there is no scientific evidence showing the side effects on oral health produced by chewing without removing aligners. In particular, the impact of CAT on oral hygiene, on cavities’ development and enamel demineralization should be further studied. Moreover, another factor to be considered is the potential distortion of aligner, even though 88.3% of the sample denied noticing any deformation of oral appliances. However, these limitations did not take into consideration the different food consistencies and the difference in chewing difficulty. The purpose of our study was to create a scale that would assess the amount of discomfort during the mastication of diverse food and the amount of residues accumulated in the oral cavity when patients eat without removing orthodontic aligners.

This study showed that when eating with aligners, certain foods can be chewed with a level of difficulty close to the lowest values on the numerical scale considered. The results demonstrated that yogurt and ice cream (and other foods of similar consistency) are the best options among chewable foods with aligners. However, in an unexpected way, fish, mozzarella and boiled vegetables also did not show a high level of difficulty. This can be explained by the fact that aligners reproduce the anatomy of teeth and so the capability of cusps to shred is partially maintained. Chewing gum is to be avoided because it can adhere to aligners and impede proper mastication. Therefore, it is conceivable and reasonable to say that chewing some of the aforementioned foods is completely compatible with the use of aligners and does not cause discomfort of significant importance. By looking at the residue values reported by the patients, it is hypothesized that these may be the main cause of discomfort when eating with aligners. However, we should note that yogurt and foods of similar consistency leave very little residue. On the other hand, crumbly foods such as cookies result in a high amount of residue.

Other studies reported that aligners do not produce a high level of discomfort while eating, because until now, the indication is to remove them before meals, but simultaneously, it was shown that discomfort caused by fixed devices during eating is quite significant. Despite the last affirmation, fixed devices are still adopted and chosen by a significant number of young and adult patients. In this line of thought, it is reasonable to affirm that the level of difficulty to chew with aligners should be assessed in comparison with the one determined by brackets [16]. 

The presence of orthodontic device fosters an increase in food residues and bacteria, which over time may lead to dental cavities or worsen any pre-existing periodontal disease [17,18]. It has been demonstrated that white spot lesions (WSL) that formed or worsened in the fixed appliance group were smaller in size but resulted in more demineralization, compromising the enamel surfaces more than WSL caused by CAT [19].

It is important to brush the inside of the aligners with water and toothpaste every time the teeth are brushed, being especially careful around the attachment wells and cusp-tip regions [20]. The protected environment of a clear aligner restricts saliva flow, therefore eliminating saliva’s natural cleaning, buffering, and remineralizing effects. Furthermore, the tongue, cheeks, and lips’ regular cleaning processes are disrupted, which promotes the growth and trapping of plaque beneath the appliances [21]. 

The environment of the internal surface of clear aligners may negatively impact enamel health if subjects do not regularly clean their aligners. According to the literature, on the internal surface of clear aligners, the pH values significantly decreased after 12 and 24 h of use, because of the reduction in microbial diversity with an increase in Granulicatella, Porphyromonas, Prevotella, Haemophilus, Streptococcus, and Acinetobacter. These bacteria could be the cause of the lower level of Ph. Consequently, it is recommended to clean aligners after 12 h of use or at least within 24 h of use [22].

It is conceivable that attachments can cause less retention of dental plaque and food residues than brackets, but according to patients, attachments without aligners scratch soft tissues like the tongue, mucosa and lips [23]. Hence, in cases where a great number of attachments are used, patients may feel less discomfort eating with the aligners on. 

The presence of a great number of attachments increases the fitting and retention of aligners, which is an important factor to avoid misfitting during mastication, even though 69.2% of the respondents affirmed not to perceive any movement of aligners. In the same way, chewing with aligners reduces the possibility of attachments’ loss because of the lower mechanical pressure applied on them. Furthermore, decreasing the frequency of taking out aligners can diminish the possibility of misplacement of the engagers [24].

Another aspect to take into consideration is the abrasion and wear of clear aligners caused by the consumption of acidic beverages and enzymatic functions elaborated in the oral cavity, potentially resulting in an increase in the release of microplastics. This is particularly noticeable in areas such as the cusp tips, which are exposed to the pressure exerted by opposing teeth during chewing [25].

Overall, a great portion of the patients (79.2%) of the sample affirmed that they would eat with the aligner if that would allow them to speed up the timing of therapy. This means that having meals using aligners is bearable and can be offered to patients as a solution to reduce the therapy’s time and discomfort. Some patients reported that taking the aligners out before meals and placing them back right after would create some social embarrassment [26]. The presence of residues remains an issue, even more so because half of the sample declared that drinking water was not enough to decrease the residue load. 

Overall, it is important to mention some limitations of this study. All the limits of an anonymous questionnaire were considered. By its very nature, a questionnaire involves personal perceptions, personal knowledge, and opinions on a certain subject. It does not include objective measurements, such as clinical measurements or physical tests on aligners structural integrity. It can be useful in future studies to introduce mechanical testing of aligners after exposure to different types of foods.

Some patients might not be aware of the significance of some questions or the intended meaning of some answers. This study has limitations caused by the complexity of the perception of chewing difficulty and food residues. According to the Checklist for Reporting Results of Internet E-Surveys (CHERRIES), the result of a web survey using an anonymous questionnaire cannot be evaluated like a certain result, but it can only be considered as a hypothesis that should be validated in a more thoroughly checked study. However, this study cannot be considered a web survey because it was widespread through clinical practices. Another possible limitation is the single geographic location instead of a multicentric worldwide study. The absence of a control group can be a weakness of this study. 

## 5. Conclusions

According to the results of this study, using clear aligners when eating does not result in high level of difficulty in chewing food and in a great amount of residue. Using clear aligners while chewing foods like yogurt, ice cream, soft cheese, bread, rice, etc., can be possible and 24 h aligner use can be accepted because it may shorten the duration of orthodontic treatment, which is advantageous for the patient and the orthodontist. 

However, it is also important to consider the disadvantages. Specifically, it has been observed that some foods and textures may cause discomfort for the patient, even though 69.2% of the respondents claimed not to have noticed any aligner movement. This might be mitigated by adding more attachments to the oral device, which would boost its retention. 

Therefore, to accomplish optimal chewing with aligners, it is advised to choose meals like yogurt, ice cream, soft cheese, bread, rice, or foods of similar consistency. It is suggested to avoid hard or chewy foods that might be uncomfortable and result in more residues.

Considering the lack of other studies regarding the topic, further research including a wide sample size and other possible ways to analyze the capability to chew food with the aligners should be conducted.

## Figures and Tables

**Table 1:** Answers to the questionnaire.

			Frequency	Percentage	Valid Percentage	Cumulative Percentage
Did you notice some movements of the aligners during chewing?	Valid	No	166	69.2	69.2	69.2
		Yes	74	30.8	30.8	100.0
		Total	240	100.0	100.0	-
Have the aligners ever broken or deformed during chewing?	Valid	No	212	88.3	88.3	89.2
		Yes	26	10.8	10.8	100.0
		Total	240	100.0	100.0	-
If you feel food residues after chewing, is drinking enough to remove them?	Valid	No	126	52.5	52.5	52.5
		Yes	114	47.5	47.5	100.0
		Total	240	100.0	100.0	-
Did you notice any difference in the length of your lunch with and without aligners?	Valid	No	119	49.6	49.6	50.0
		Yes	120	50.0	50.0	100.0
		Total	240	100.0	100.0	-
If eating with aligners was proven to speed up treatment, would you continue to eat with aligners?	Valid	No	50	20.8	20.8	20.8
		Yes	190	79.2	79.2	100.0
		Total	240	100.0	100.0	-

**Table 2:** Cronbach’s alpha: the reliability test using the test–retest method has a correlation coefficient greater than 0.9 for all items (the cutoff is 0.8) with excellent significance and excellent internal consistency with an alpha greater than 0.9.

Question	Coefficient	Significance
Number of attachments	0.998	*p* < 0.001
Are the aligners adherent?	0.921	*p* < 0.001
Bread: [Difficulty]	0.981	*p* < 0.001
Bread: [Residues]	0.955	*p* < 0.001
Yogurt or ice cream: [Difficulty]	0.967	*p* < 0.001
Yogurt or ice cream: [Residues]	0.879	*p* < 0.001
Rice: [Difficulty]	0.971	*p* < 0.001
Rice: [Residues]	0.96	*p* < 0.001
Meat or shellfish: []	0.841	*p* < 0.001
Meat or shellfish: [Residues]	0.927	*p* < 0.001
Boiled vegetables: [Difficulty]	0.969	*p* < 0.001
Boiled vegetables: [Residues]	0.932	*p* < 0.001
Pasta: [Difficulty]	0.99	*p* < 0.001
Pasta: [Residues]	0.982	*p* < 0.001
Chewing gum: [Difficulty]	0.937	*p* < 0.001
Chewing gum: [Residues]	0.964	*p* < 0.001
Biscuits or breadsticks or crackers: [Difficulty]	0.96	*p* < 0.001
Biscuits or breadsticks or crackers: [Residues]	0.993	*p* < 0.001
Fish: [Difficulty]	0.983	*p* < 0.001
Fish: [Residues]	0.979	*p* < 0.001
Meatballs or Hamburgers: [Difficulty]	0.979	*p* < 0.001
Meatballs or Hamburgers: [Residues]	0.97	*p* < 0.001
Cold cuts or ham: [Difficulty]	0.975	*p* < 0.001
Cold cuts or ham: [Residues]	0.936	*p* < 0.001
Mozzarella or soft cheese: [Difficulty]	0.962	*p* < 0.001
Mozzarella or soft cheese: [Residues]	0.986	*p* < 0.001
Chips or popcorn or nuts or peanuts: [Difficulty]	0.966	*p* < 0.001
Chips or popcorn or nuts or peanuts: [Residues]	0.98	*p* < 0.001
Salad: [Difficulty]	0.989	*p* < 0.001
Salad: [Residues]	0.979	*p* < 0.001
Sandwiches: [Difficulty]	0.938	*p* < 0.001
Sandwiches: [Residues]	0.925	*p* < 0.001

**Table 3:** Variables analyzed in the questionnaire.

Personal Details	Aligners Details	Chewing Function	Tested Foods
Age Gender Educational background If it was proven that eating while wearing the aligners accelerates orthodontic treatment, would you be willing to continue doing so?	Number of attachments Adherence of aligners	Did you notice any difference between wearing and not wearing the aligners during lunch? Did you feel the aligners moving during chewing? Have the aligners broken or deformed during chewing? If you found food residues after chewing, was drinking sufficient to remove them?	Bread Yogurt or ice cream Rice Meat or clams Boiled vegetables Pasta Pizza Chewing gum Biscuits or breadsticks or crackers Fish Meatballs or hamburgers Processed meats or ham Mozzarella or soft cheese Chips or popcorn or nuts or peanuts Salad Sandwiches

**Table 4:** Demographic data.

	Age	Sex	Qualification	Number of Attachments?	Are the Aligners Tight?
N	218	233	234	207	229
Not responding	22	7	6	33	11
Average	30.0			10.1	4.59
Median	28.0			12	5
Standard Deviation	9.22			7.84	0.626
Minimum	12			0	2
Maximum	61			28	5

**Table 5:** Level of chewing difficulty.

Difficulty	Valid	Median		Percentiles	
			25	50	75
Bread	157	3	2	3	5.5
Yogurt or ice cream	188	0	0	0	2.75
Rice	166	3	1	3	5
Meat or clams	164	5	2	5	8
Boiled vegetables	170	2	1	2	4
Pasta	165	3	2	3	5
Pizza	121	4	2	4	6
Chewing gum	152	7	1.25	7	10
Biscuits or breadsticks or crackers	160	3	1	3	5
Fish	161	2	1	2	5
Meatballs or hamburger	162	3	1	3	5
Sausage or ham	173	4	2	4	6
Mozzarella or soft cheese	170	2	0	2	4
Salad	167	3	2	3	6
Sandwiches	161	3	2	3	5
Chips or popcorn or nuts or peanuts	160	4	2	4	6

**Table 6:** Amount of residue.

Residues	N	Minimum	Maximum	Average	Standard Deviation
Bread	155	0	10	3.62	2.356
Yogurt or ice cream	188	0	10	1.86	2.279
Rice	165	0	8	3.10	2.117
Meat or clams	165	0	10	4.25	2.531
Boiled vegetables	170	0	10	3.20	2.480
Pasta	165	0	10	3.27	2.069
Pizza	121	0	10	3.83	2.147
Chewing gum	153	0	10	5.31	3.769
Biscuits or breadsticks or crackers	159	0	10	4.65	2.521
Fish	161	0	10	3.02	2.191
Meatballs or hamburger	163	0	10	3.37	2.085
Sausage or ham	174	0	10	3.38	2.053
Mozzarella or soft cheese	170	0	10	2.72	2.293
Chips or popcorn or nuts or peanuts	161	0	10	5.37	2.578
Salad	167	0	10	3.93	2.456
Sandwiches	162	0	10	3.49	2.010
Number of valid cases (listwise)	91				

**Table 7:** Pearson’s test for chewing difficulty.

Difficulty		Biscuits or Breadsticks or Crackers	Fish	Meatballs or Hamburger	Sausage or Ham	Mozzarella or Soft Cheese	Chips or Popcorn or Nuts or Peanuts	Salad	Sandwiches
Bread	Pearson’s correlation	0.699	0.710	0.735	0.289	0.594	0.752	0.553	0.721
	Sign.	0.000	0.000	0.000	0.000	0.000	0.000	0.000	0.000
	N	146	143	144	143	144	145	144	145
Yogurt or ice cream	Pearson’s correlation	0.438	0.648	0.631	0.531	0.733	0.454	0.570	0.682
	Sign.	0.000	0.000	0.000	0.000	0.000	0.000	0.000	0.000
	N	149	148	150	159	153	148	156	151
Rice	Pearson’s correlation	0.653	0.633	0.722	0.382	0.627	0.640	0.638	0.610
	Sign.	0.000	0.000	0.000	0.000	0.000	0.000	0.000	0.000
	N	146	142	146	145	146	145	150	146
Meat or clams	Pearson’s correlation	0.423	0.307	0.424	0.512	0.039	0.391	0.479	0.417
	Sign.	0.000	0.000	0.000	0.000	0.635	0.000	0.000	0.000
	N	143	142	143	144	148	143	144	142
Boiled vegetables	Pearson’s correlation	0.616	0.768	0.796	0.419	0.763	0.548	0.567	0.666
	Sign.	0.000	0.000	0.000	0.000	0.000	0.000	0.000	0.000
	N	147	145	145	145	146	145	149	144
Pasta	Pearson’s correlation	0.725	0.776	0.857	0.507	0.689	0.688	0.589	0.711
	Sign.	0.000	0.000	0.000	0.000	0.000	0.000	0.000	0.000
	N	145	143	149	146	147	142	145	143
Pizza	Pearson’s correlation	0.753	0.797	0.828	0.394	0.636	0.703	0.550	0.682
	Sign.	0.000	0.000	0.000	0.000	0.000	0.000	0.000	0.000
	N	103	103	103	106	107	102	104	105
Chewing gum	Pearson’s correlation	0.552	0.548	0.604	0.12	0.501	0.692	0.460	0.502
	Sign.	0.000	0.000	0.000	0.151	0.000	0.000	0.000	0.000
	N	142	142	143	144	144	142	142	141
Biscuits or breadsticks or crackers	Pearson’s correlation	1	0.759	0.768	0.302	0.499	0.798	0.512	0.625
	Sign.		0.000	0.000	0.000	0.000	0.000	0.000	0.000
	N	160	142	141	142	144	144	141	142
Fish	Pearson’s correlation	0.759	1	0.903	0.544	0.738	0.696	0.555	0.675
	Sign.	0.000		0.000	0.000	0.000	0.000	0.000	0.000
	N	142	161	142	146	144	141	145	138
Meatballs or hamburger	Pearson’s correlation	0.768	0.903	1	0.518	0.745	0.701	0.533	0.729
	Sign.	0.000	0.000		0.000	0.000	0.000	0.000	0.000
	N	141	142	162	150	145	142	143	142
Sausage or ham	Pearson’s correlation	0.302	0.544	0.518	1	0.463	0.305	0.675	0.564
	Sign.	0.000	0.000	0.000		0.000	0.000	0.000	0.000
	N	142	146	150	173	149	145	150	143
Mozzarella or soft cheese	Pearson’s correlation	0.499	0.738	0.745	0.463	1	0.516	0.566	0.675
	Sign.	0.000	0.000	0.000	0.000		0.000	0.000	0.000
	N	144	144	145	149	170	147	149	144
Chips or popcorn or nuts or peanuts	Pearson’s correlation	0.798	0.696	0.701	0.305	0.516	1	0.588	0.585
	Sign.	0.000	0.000	0.000	0.000	0.000		0.000	0.000
	N	144	141	142	145	147	160	143	145
Salad	Pearson’s correlation	0.512	0.555	0.533	0.675	0.566	0.588	1	0.673
	Sign.	0.000	0.000	0.000	0.000	0.000	0.000		0.000
	N	141	145	143	150	149	143	167	145
Sandwiches	Pearson’s correlation	0.625	0.675	0.729	0.564	0.675	0.585	0.673	1
	Sign.	0.000	0.000	0.000	0.000	0.000	0.000	0.000	
	N	142	138	142	143	144	145	145	161

**Table 8:** Pearson’s test for residues.

Residues		Biscuits or Breadsticks or Crackers	Fish	Meatballs or Hamburger	Sausage or Ham	Mozzarella or Soft Cheese	Chips or Popcorn or Nuts or Peanuts	Salad	Sandwiches
Bread	Pearson’s correlation	0.447	0.637	0.677	0.517	0.553	0.408	0.589	0.618
	Sign.	0.000	0.000	0.000	0.000	0.000	0.000	0.000	0.000
	N	143	141	143	142	142	144	142	144
Yogurt or ice cream	Pearson’s correlation	0.279	0.494	0.500	0.309	0.500	0.311	0.425	0.450
	Sign.	0.001	0.000	0.000	0.000	0.000	0.000	0.000	0.000
	N	148	148	151	160	153	149	156	152
Rice	Pearson’s correlation	0.575	0.634	0.579	0.562	0.606	0.548	0.572	0.513
	Sign.	0.000	0.000	0.000	0.000	0.000	0.000	0.000	0.000
	N	144	141	146	145	145	145	149	146
Meat or clams	Pearson’s correlation	0.335	0.541	0.534	0.551	0.523	0.436	0.500	0.537
	Sign.	0.000	0.000	0.000	0.000	0.000	0.000	0.000	0.000
	N	143	143	145	146	149	145	145	144
Boiled vegetables	Pearson’s correlation	0.481	0.701	0.674	0.475	0.668	0.461	0.582	0.616
	Sign.	0.000	0.000	0.000	0.000	0.000	0.000	0.000	0.000
	N	146	145	146	146	146	146	149	145
Pasta	Pearson’s correlation	0.557	0.742	0.780	0.652	0.656	0.500	0.577	0.678
	Sign.	0.000	0.000	0.000	0.000	0.000	0.000	0.000	0.000
	N	144	143	150	147	147	143	145	144
Pizza	Pearson’s correlation	0.408	0.676	0.750	0.585	0.705	0.448	0.481	0.614
	Sign.	0.000	0.000	0.000	0.000	0.000	0.000	0.000	0.000
	N	102	103	103	106	107	102	104	106
Chewing gum	Pearson’s correlation	0.318	0.565	0.479	0.500	0.621	0.537	0.523	0.466
	Sign.	0.000	0.000	0.000	0.000	0.000	0.000	0.000	0.000
	N	142	143	145	146	145	144	143	143
Biscuits or breadsticks or crackers	Pearson’s correlation	1	0.502	0.562	0.561	0.444	0.736	0.534	0.493
	Sign.		0.000	0.000	0.000	0.000	0.000	0.000	0.000
	N	159	141	141	142	143	144	140	142
Fish	Pearson’s correlation	0.502	1	0.749	0.664	0.676	0.430	0.648	0.687
	Sign.	0.000		0.000	0.000	0.000	0.000	0.000	0.000
	N	141	161	143	147	144	142	145	139
Meatballs or hamburger	Pearson’s correlation	0.562	0.749	1	0.568	0.662	0.480	0.531	0.698
	Sign.	0.000	0.000		0.000	0.000	0.000	0.000	0.000
	N	141	143	163	152	146	144	144	144
Sausage or ham	Pearson’s correlation	0.561	0.664	0.568	1	0.536	0.444	0.609	0.596
	Sign.	0.000	0.000	0.000		0.000	0.000	0.000	0.000
	N	142	147	152	174	150	146	151	145
Mozzarella or soft cheese	Pearson’s correlation	0.444	0.676	0.662	0.536	1	0.395	0.456	0.640
	Sign.	0.000	0.000	0.000	0.000		0.000	0.000	0.000
	N	143	144	146	150	170	147	149	145
Chips or popcorn or nuts or peanuts	Pearson’s correlation	0.736	0.430	0.480	0.444	0.395	1	0.593	0.523
	Sign.	0.000	0.000	0.000	0.000	0.000		0.000	0.000
	N	144	142	144	146	147	161	144	147
Salad	Pearson’s correlation	0.534	0.648	0.531	0.609	0.456	0.593	1	0.673
	Sign.	0.000	0.000	0.000	0.000	0.000	0.000		0.000
	N	140	145	144	151	149	144	167	146
Sandwiches	Pearson’s correlation	0.493	0.687	0.698	0.596	0.640	0.523	0.673	1
	Sign.	0.000	0.000	0.000	0.000	0.000	0.000	0.000	
	N	142	139	144	145	145	147	146	162

## Data Availability

The raw data supporting the conclusions of this article will be made available by the authors on request due to privacy.
